# Is the Non-Coding RNA miR-195 a Biodynamic Marker in the Pathogenesis of Head and Neck Squamous Cell Carcinoma? A Prognostic Meta-Analysis

**DOI:** 10.3390/jpm13020275

**Published:** 2023-01-31

**Authors:** Mario Dioguardi, Francesca Spirito, Giorgia Apollonia Caloro, Lorenzo Lo Muzio, Stefania Cantore, Andrea Ballini, Salvatore Scacco, Annarita Malcangi, Salvatore Sembronio, Eliano Cascardi, Roberto Arrigoni, Michele Di Cosola, Riccardo Nocini

**Affiliations:** 1 Department of Clinical and Experimental Medicine, University of Foggia, Via Rovelli 50, 71122 Foggia, Italy; 2 Unità Operativa Nefrologia e Dialisi, Presidio Ospedaliero Scorrano, ASL (Azienda Sanitaria Locale) Lecce, Via Giuseppina Delli Ponti, 73020 Scorrano, Italy; 3 Independent Researcher, Sorriso & Benessere-Ricerca e Clinica, 70129 Bari, Italy; 4 Department of Precision Medicine, University of Campania “Luigi Vanvitelli”, 80138 Naples, Italy; 5 Department of Translational Biomedicine and Neuroscience (DiBraiN), University of Bari “Aldo Moro”, 70121 Bari, Italy; 6 Public Local Health Company (Azienda Sanitaria Locale, ASL), B.A.T, 76125 Trani, Italy; 7 Maxillofacial Surgery Department, Academic Hospital of Udine, Department of Medical Science, University of Udine, P.le Kolbe 4, 33100 Udine, Italy; 8 Department of Medical Sciences, University of Turin, 10100 Turin, Italy; 9 CNR Institute of Biomembranes, Bioenergetics and Molecular Biotechnologies (IBIOM), 70124 Bari, Italy; 10 Unit of Otolaryngology-Head and Neck Department, University of Verona, 37126 Verona, Italy

**Keywords:** miR-195, microRNA, head and neck squamous cell carcinoma (HNSCC), noncoding RNA, oral cancer, trial sequential analysis

## Abstract

Head and neck squamous cell carcinoma (HNSCC) represents a heterogeneous group of neoplasms whose histological derivation comes from the mucous membranes lining the epithelium: the oral cavity, the larynx, the hypopharynx, the nasopharynx, and the oropharynx. The etiopathogenetic mechanisms involving tumor genesis including the alteration of cell proliferation, apoptosis, invasion, migration, and death may involve alterations in the expression of microRNA (miR). To date there have been no systematic reviews with meta-analysis conducted specifically on the role of miR-195 in HNSCC; therefore, our hypothesis was to evaluate if the aberrant expression of miR-195 in HNSCC tissues may represent a prognostic biomarker of survival through the hazard ratio (HR) and relative risk (RR) analysis. The systematic review was designed according to the PRISMA indications; in total, three electronic databases were consulted (PubMed, Scopus, Cochrane Central Trial) including Google Scholar and the gray literature, and a combination of keywords was used such as miR-195 AND HNSCC, microRNA AND HNSCC and miR-195. The meta-analysis and trial sequential analysis were performed using RevMan 5.41 software and TSA software (Cochrane Collaboration, Copenhagen, Denmark). This search identified 1592 articles and, at the end of the selection process, three articles were included. The results of the meta-analysis reported an aggregated risk ratio for overall survival (OS) between the expression of miR-195 at the highest and lowest of 0.36 and 6, respectively, 95% CI: [0.25, 0.51]. Heterogeneity was evaluated through Chi^2^ = 0.05 df = 2 (*p* = 0.98) and the Higgins index I^2^ = 0%. The test for the overall effect was Z = 5.77 (*p* < 0.00001). The forest plot was in favor of higher OS in patients with high miR-195 expression.

## 1. Introduction

Head and neck squamous cell carcinoma (HNSCC) embodies a heterogeneous group of neoplasms whose histological derivation comes from the lining epithelium of the mucous membranes: the oral cavity (oral squamous cell carcinoma OSCC), the larynx (squamous cell carcinoma of the larynx, LSCC), the hypopharynx (squamous cell carcinoma of the hypopharynx, HSCC), the nasopharynx (nasopharyngeal carcinoma, NPC), and the oropharynx (squamous cell carcinoma of the oropharynx, OPSCC) [1].

HNSCC represents the sixth cancer by incidence with 370,000 new cases per year, with an estimated 5-year average survival for stages 3 and 4 of 30% [2,3].

The recognized risk factors for HNSCC are smoking and alcohol, which have a synergistic effect, furthermore, for laryngeal carcinomas, there is a correlation with HPV specifically for subtypes 16–18; generally, LSCC HPV + has a better prognosis by responding more effectively to radiotherapy [4,5]. 

The etiopathogenetic mechanisms involving tumor genesis including alterations in cell/stem cell proliferation, apoptosis, invasion, migration, and death may involve alterations in the expression of microRNA (miR) [6,7,8,9,10,11,12,13,14,15].

MicroRNA are a small non-coded RNA sequence of 18–22 nucleotides whose function is to regulate the gene expression of genes essential for the performance of physiological and pathological cellular activities [16], which can be found in tumor or precancerous tissues as well as upregulated (miR-21, miR-27, miR-31, miR-93, miR-134, miR-146, miR-155, miR-196a, miR-211, miR-218, miR-222, miR-372 and miR-373) and downregulated (let-7, miR-26a, miR-99a-5p, miR-137, miR-139, miR-143, miR-184, miR-375 and miR-195) [17].

Many miR have shown prognostic survival capabilities with potential as a biomarker. In the head and neck area, the main miR associated with a potential biomarker is miR-21 [18], which would be upregulated in tumor tissues whose overexpression is potentially associated with a worsening of prognosis; similarly, other upregulated miRs have also been investigated, and among these, the miR-31 [19] and miR-155 [20], among the downregulated miRs, the focus has mainly on the Let-7 microRNA family. Recently, some interesting studies have turned toward miR-195, which would be downregulated in the tissues of many cancers including bladder cancer, breast, stomach, lung, bone, and liver.

Its localization is on chromosome 17p13.1 and its mature form has the sequence of 5′ AGCAGCACAGAAAUAUUGGC 3′ and besides, MiR-195 performs various regulatory functions in the cell cycle, especially between the transition phases G2/M and G1/S, also promoting apoptosis by inhibiting the expression of Bcl-2 [21].

Recent studies conducted in close proximity of the head and neck area associated the downregulation of miR-195 in cancer tissues with a worsening of the prognostic survival indices. In 2014, Sun et al. found that aberrant expression of miR-195 could act as a promising poor prognostic biomarker for esophageal squamous cell carcinoma (ESCC) [22], and analogous results were found for LSCC. In 2017, Shuang [23], on a cohort of 122 patients with laryngeal carcinoma identified a relative risk (RR) of death at the end of the follow-up period between high and low expression of 0.358, indicating that there is a greater risk of death in patients with a downregulation in carcinoma tissues of miR-195 [23].

To date, there has been no systematic review with meta-analyses conducted specifically on the role of miR-195 in HNSCC because of the presence in the literature of recent studies conducted on miR-195; by aggregating the results, it is possible to clearly determine, in our hypothesis, if the aberrant expression of miR-195 in HNSCC tissues may represent a prognostic biomarker of survival through the hazard ratio (HR) and RR analysis.

## 2. Materials and Methods

### 2.1. Protocol

The planning of this systematic review and meta-analysis was performed according to the recommendations of the Cochrane Handbook for Systematic Reviews of Interventions, and following the indications of the PRISMA (Preferred Reporting Items for Systematic Reviews and Meta-Analysis) [18,19,20,24,25,26,27] The review protocol was previously registered on INPLASY (International Platform of Registered Systematic Review and Meta-analysis Protocols) with registration number INPLASY202240150 and the DOI is 10.37766/inplasy2022.4.0150.

### 2.2. Eligibility Criteria

The search for sources was directed toward all randomized control trials, prospective non-randomized studies, and retrospective studies that investigated the role of miR-195 in HNSCC, with a clear reference to the prognostic survival indices in correlation with expression. Regarding miR-195, particularly the HR and RR values between high and low miR-195 expression had to be indicated.

The PICO question formulated was the following: what is the RR and HR in the prognostic indices of survival among HNSCC patients with high tissue miR-195 expression compared to those with low expression? The different points studied were: (P) participants (patients with HNSCC), (I) intervention (impaired expression of miR-195in HNSCC), (C) control (patients with HNSCC who have low expression of miR-195), and (O) outcome (difference in death risk of survival prognosis between patients with low and high miR-195 expression in HNSCC).

The exclusion criteria for the systematic review were the following: (1) Not written in English; (2) not reporting the HR or the RR, or alternatively not clearly reporting the number of HNSCC with high and low expression with the number of deaths at the end of the follow-up period (for the calculation of the RR); (3) high risk or bias; (4) literature reviews (considered only as sources of information and bibliographic references); and (5) case series and case reports.

Therefore, it was decided to include studies that investigated miR-195 in relation to HNSCC that had the value of RR or HR, or that can be calculated with statistical methods; in relation to HR, prognostic survival indices were taken into consideration such as overall survival (OS), disease free survival (DFS), recurrence free survival (RFS), progression free survival (PFS), and cancer specific survival (CSS), which reported the numerical value (HR), or the Cox regression, or the Kaplan–Meier survival curves.

### 2.3. Sources of Information, Research, and Selection

The research of the studies involved two independent reviewers (M.D. and S.C.). The research and selection phase of the articles was carried out in three phases: in the first phase, the inclusion and exclusion criteria, the databases, the keywords to be used and the period in which to conduct the search were jointly decided; in the second phase, we proceeded separately to perform the research and selection of the studies with the removal of the overlaps using reference management software such as EndNote 8.0 with the inclusion of the studies; and in the third phase, we proceeded to compare the included studies and resolve any conflicts between the two reviewers with the help, if necessary, of a third reviewer (A.B.) to decide on doubtful situations.

The keywords used were miR-195 AND HNSCC, microRNA AND HNSCC, LSCC AND miR-195, OSCC AND miR-195, OPSCC AND miR-195, HSCC AND miR-195.

The research was conducted on two databases: SCOPUS and PubMed, and a registry: Cochrane Central Trial; in addition, Google Scholar (keywords miR-195), gray literature sources such as Open Gray (keywords miR), and the bibliographic references of previous systematic reviews on miR and HNSCC were consulted,

Specifically, below are all the keywords used in PubMed: (((((((((((“opscc”[All Fields] OR “opsccs”[All Fields]) AND “miR-195”[All Fields]) OR “HSCC”[All Fields]) AND “miR-195”[All Fields]) OR “LSCC”[All Fields]) AND “miR-195”[All Fields]) OR “OSCC”[All Fields]) AND “miR-195”[All Fields]) OR “miR-195”[All Fields]) AND (“hnsccs”[All Fields] OR “squamous cell carcinoma of head and neck”[MeSH Terms] OR (“squamous”[All Fields] AND “cell”[All Fields] AND “carcinoma”[All Fields] AND “head”[All Fields] AND “neck”[All Fields]) OR “squamous cell carcinoma of head and neck”[All Fields] OR “hnscc”[All Fields])) OR (“micrornas”[All Fields] OR “ microRNA “[MeSH Terms] OR “ microRNAs “[All Fields] OR “ microRNA “[All Fields])) AND (“hnsccs”[All Fields] OR “squamous cell carcinoma of head and neck”[MeSH Terms] OR (“squamous”[All Fields] AND “cell”[All Fields] AND “carcinoma”[All Fields] AND “head”[All Fields] AND “neck”[All Fields]) OR “squamous cell carcinoma of head and neck”[All Fields] OR “hnscc”[All Fields]); Translations OPSCC: “opscc”[All Fields] OR “opsccs”[All Fields]; HNSCC: “hnsccs”[All Fields] OR “squamous cell carcinoma of head and neck”[MeSH Terms] OR (“squamous”[All Fields] AND “cell”[All Fields] AND “carcinoma”[All Fields] AND “head”[All Fields] AND “neck”[All Fields]) OR “squamous cell carcinoma of head and neck”[All Fields] OR “hnscc”[All Fields]; microRNA: “microRNA’s”[All Fields] OR “microRNAs”[MeSH Terms] OR “microRNAs”[All Fields] OR “microRNA”[All Fields]; HNSCC: “hnsccs”[All Fields] OR “squamous cell carcinoma of head and neck”[MeSH Terms] OR (“squamous”[All Fields] AND “cell”[All Fields] AND “carcinoma”[All Fields] AND “head”[All Fields] AND “neck”[All Fields]) OR “squamous cell carcinoma of head and neck”[All Fields] OR “hnscc”[All Fields].

The record search was completed on 30 September 2022, and a final update on the search was carried out on 5 October 2022.

### 2.4. Data Collection Process and Data Characteristics

The data extraction process was performed by the two reviewers after the article inclusion phase and reported in two tables to be compared. The type of data to be extracted was agreed in advance before the article selection phase and was related to the first author, date of publication, the country where the study was conducted, the type of study, the follow-up period, the type of HNSCC, any other targets investigated the cut-off value between low and high miR-195 expression on the number of deaths for the two groups, the number of patients, the RR value, and the HR value between high and low expression, if present for the various prognostic survival indices.

The possibility of extracting the HR values between high and low expression if the Kaplan–Meier survival curves were present were also evaluated following the method of Tierney et al. [28].

### 2.5. Risk of Bias in Individual Studies, Summary Measures, Summary of Results, Risk of Bias between Studies, Additional Measures

The risk of bias in the individual studies was assessed by an author (MD), as a tool for the evaluation parameters derived from the reporting recommendations for prognostic studies of markers (REMARK) [29]. Studies with a high risk of bias were excluded from the meta-analysis. The risk of bias between the studies, on the other hand, was assessed through the heterogeneity indices (Higgins index I^2^) and graphically through the visual analysis of the overlap of the confidence intervals in the various forest plots and through the funnel plot; the asymmetry of the funnel plot was used for a publication bias assessment. The possibility of carrying out a sensitivity analysis was also evaluated to identify and exclude the source of heterogeneity; furthermore, trial sequential analysis (TSA) was performed for the evaluation of the statistical power of the meta-analysis and the GRADE for the quality of the evidence.

For the meta-analysis, Reviewer Manager 5.4 software (Copenhagen Trial Unit, Center for Clinical Intervention Research, Copenhagen, Denmark) was used. The online software GRADE pro-Guideline Development Tool (GRADEpro GDT, Evidence Prime, Hamilton, ON, Canada) was applied to evaluate the quality of the evidence. The TSA was performed using a Java-based software, the TSA software (Copenhagen Trial Unit, Center for Clinical Intervention Research, Copenhagen, Denmark) [30].

In addition, a bioinformatic analysis was performed on the TGCA (The Cancer Genome Atlas), which presented a cohort of 512 patients with HNSCC through the Kaplan–Meier Plotter database portal (https://kmplot.com/analysis/, accessed on 20 December 2022). Specifically, the HR value between high and low miR-195 expression for OS was calculated and generated from the portal. This meta-analysis demonstrates that miR-195 is clearly downregulated in HNSCC carcinoma tissues.

## 3. Results

### 3.1. Selection of Studies

**Figure 1:**
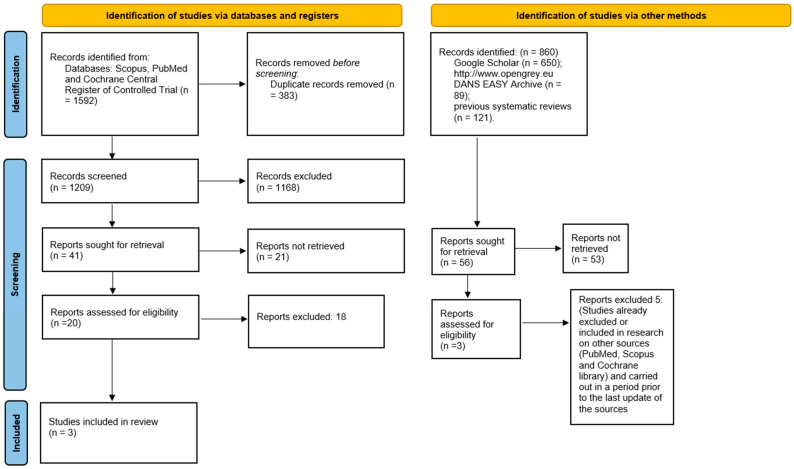
Entire selection and screening procedures are described in the PRISMA flowchart.

The search in the Scopus, PubMed databases and in the Cochrane Central Register of Controlled Trial resulted in 1592 bibliographic sources; by duplicate removal, 1209 were obtained. The articles that were potentially eligible numbered 20, of which only three fully complied with the inclusion and exclusion criteria and the related extracted data were included in the meta-analysis. Furthermore, the analysis of the gray literature (Google Scholar, Open Gray) and previous systematic reviews did not allow for the identification of further studies to be included in the meta-analysis (Figure 1). The entire procedure of identification, selection, and inclusion of the studies is indicated in the flowchart of Figure 1.

### 3.2. Data Characteristics

After the inclusion and exclusion phase of the reports, only three studies were included in the meta-analysis: Shuang et al. [23], Ding and Qi [31], and Jia et al. [32].

The extracted data described in the Materials and Methods section was included in Table 1. 

The total number of patients included in this review was 385, 304 of them with LSCC and 81 with TSCC, respectively. The number of female patients was 140 and 245 were male, in accordance with the epidemiological data of HNSCC incidence [4]; 201 of those were in stage I–II and 284 were stage III–IV, only one study reported the number of smokers, and no HPV positivity data were present. The maximum follow-up period for all three studies was approximately 60 months. There was no HR data, but the RR data were present. The included studies were prospective clinical studies, whose prognostic index investigated was mainly OS, in which in at least two cases the RR value between high and low miR-195 expression was available.

### 3.3. Risk of Bias

The risk of bias was assessed through parameters derived from REMARK. According to the REMARK guidelines, a score from 0 to 3 was considered for each factor (Table 2).

### 3.4. Meta-Analysis

Meta-analysis was performed using Reviewer Manager 5.4 (Cochrane Collaboration, Copenhagen, Denmark), software. It was possible to perform a single meta-analysis from the extracted data only on the main prognostic index OS considering the RR between high and low miR-195 expression; a fixed effects model was applied because the heterogeneity calculated through the Higgins index (I^2^) was 0%.

**Figure 2:**
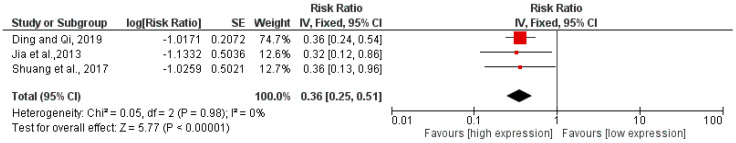
Forest plot of the fixed effects model of the meta-analysis; OS, RR = 0.36 95% CI: [0.25, 0.51]; df = degrees of freedom; I^2^ = Higgins heterogeneity index, I^2^ < 50%, heterogeneity irrelevant; *I*^2^ > 75%, significant heterogeneity; CI = confidence intervals; *p* = *p*-value; SE = standard error. The graph of each study shows the first author and publication date, log RR with confidence intervals, standard error log RR, and weight of each study expressed as a percentage. The final value is expressed in bold with the relative confidence intervals. The black line shows the position of the average value, and the light black diamond shows the measure of the average effect [23,31,32].

The results of the meta-analysis reported an aggregate RR for OS between high and low miR-195 expression of 0.36 with the relative confidence intervals (CI) [0.25 0.51], heterogeneity was evaluated through Chi^2^ = 0.05 df = 2 (*p* = 0.98) and the Higgins index I^2^ = 0%; the test for the overall effect was Z = 5.77 (*p* < 0.00001). The forest plot is in favor of higher OS in patients with high miR-195 expression (Figure 2).

### 3.5. Risk of Bias across Study, Publication Bias, Sensitivity Analysis, Subgroup Analysis

**Figure 3:**
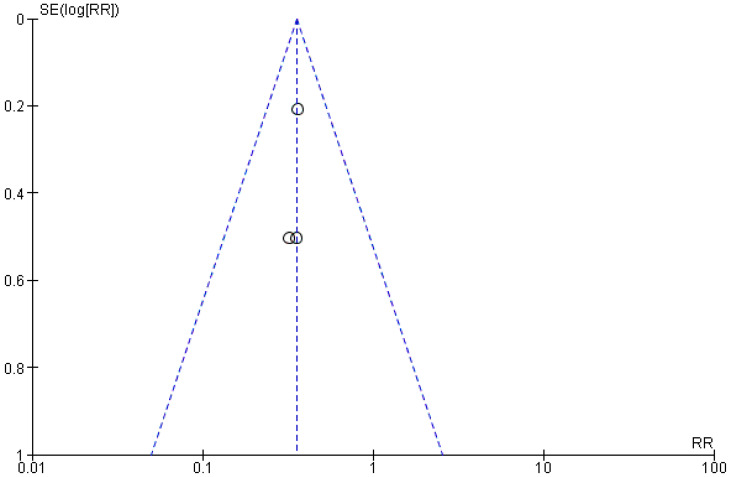
Meta-analysis funnel plot. The presence of symmetry demonstrates the potential possibility of not presenting the bias of publication.

The risk of bias between the studies was to be considered low (I^2^ = 0%) and through the visual and graphic analysis of the confidence intervals, did not emerge on the meta-analysis. An evaluation of the publication bias was also conducted, and the evaluation was performed through graphical analysis of the symmetry of the funnel plot, which appeared to be symmetric (Figure 3); to minimize the bias, a search was carried out for unpublished material in the gray literature, which also included the presence of conference papers and material that may not have been published due to the lack of significance of the results. 

Given the low number of studies included, it was decided not to perform the sensitivity and subgroup analyses. 

### 3.6. Trial Sequential Analysis, Grade, Additional Measure

Trial sequential analysis (TSA) was performed to evaluate the potency of the result of the first meta-analysis, and adjust the results to avoid type I and II errors. The program used was TSA free software.

**Figure 4:**
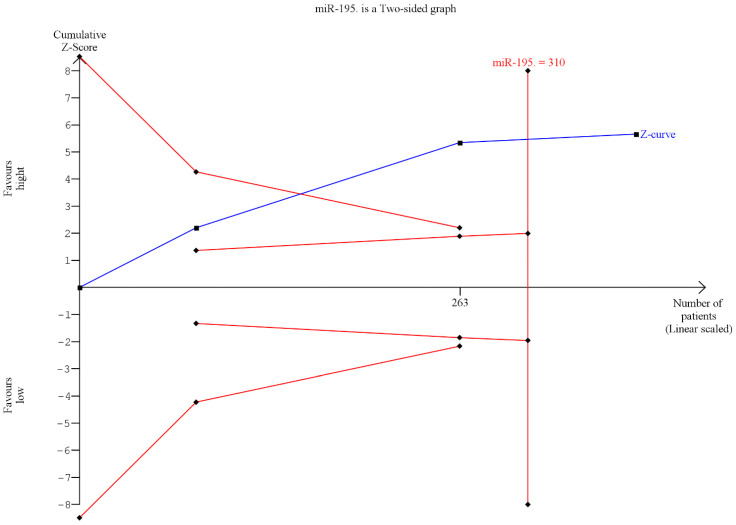
Trial sequential analysis (TSA) was performed to evaluate the potency of the result of the meta-analysis.

The results from the TSA showed that the data, despite only three studies being included, had statistical significancy; effectively it was highlighted how the blue line (Z-curve) crosses the trial sequential boundary (red inclined line). The TSA also showed that the optimal patient number of 310 was superior to the three studies (385) (Figure 4).

The authors also used GRADE pro-GDT to assess the quality of the evidence on the outcome (Table 3). The results suggested that the quality of evidence is high.

**Figure 5:**
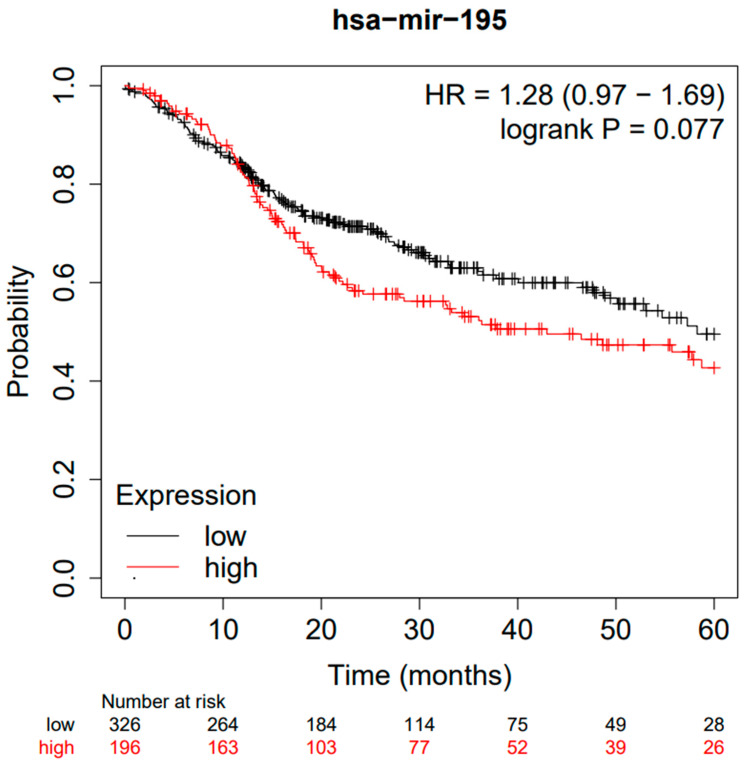
Kaplan–Meier curves according to the miR-195 expression levels for overall survival (OS) in patients with HNSCC (TGCA cohort). Kaplan–Meier curves were created by the public database and web application Kaplan–Meier Plotter (http://kmplot.com/analysis/, accessed on 20 December 2022).

**Figure 6:**
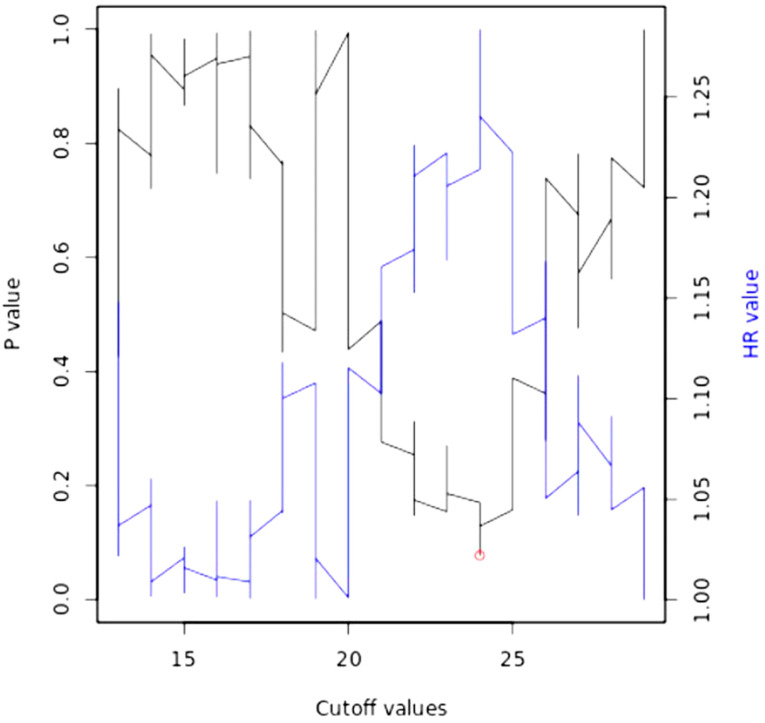
Auto cut-off plot. Significance vs. cut-off values between the lower and upper quartiles of expression.

Bioinformatics analyses were performed on the cohort of 512 HNSCC patients (TGCA) through the Kaplan–Meier Plotter database portal (https://kmplot.com/analysis/, accessed on 20 December 2022), generating the following Kaplan–Meier curve (Figure 5), between high and low miR-195 expression. A follow-up period of 60 months was set as a parameter and the cut-off was self-calculated by the portal as at 24 (fold change) (Figure 6); all the cut-off values in relation to the *p*-values are available in the Supplemental Materials.

Bioinformatics analysis of miR-195 reported an HR for OS between high and low HR expression = 1.28 (0.97–1.69) and a log rank *p*-value of 0.077 with a median survival for the low expression cohort (months) of 58.27 months and for high expression cohort (months) of 42.97.

## 4. Discussion

A systematic literature review was made with the TSA being performed on the value as a prognostic biomarker of survival of miR-195 in head and neck carcinomas. To the best of our knowledge, this systematic review represents the first meta-analysis to focus on miR-195 in HNSCC followed by TSA for the analysis of the statistical significance of the results. Three studies were included in this review, with a total of 385 patients recruited; the publication bias was either minimized by including the search on many databases including some of the gray literature and, additionally, the TSA results showed that the results of the meta-analysis had an adequate statistical power.

Several studies have confirmed that miR-195 is downregulated in carcinoma tissues, for example, the most recent studies of Li et al. [33], found that it was downregulated in squamous cell carcinoma of the esophagus, while a recent review of the literature conducted by Xu et al. [34], they found that it downregulated in very many solid tumors, indicating that its overexpression is generally associated with a tumor growth inhibition effect.

For head and neck HNSCCs, Ding and Qi [34], reported that miRNA-195 expression was downregulated in LSCC tissue, and that low miRNA-195 expression may be related to laryngeal cancer invasion and metastasis, a poor prognosis indicator [31]. Even for LSCC, there exists unfavorable prognosis data, according to Shuang et al. [23].

The results obtained for the LSCCs are also consistent with the OSCCs, and in particular, with the TSCCs: Jia et al. [32] confirmed these data in a sample of the homogeneous patient population in which the expression of miR-195 was downregulated in about 80% of tumor tissues compared to the adjacent healthy tissues [32].

All three studies reported similar RR values between high and low expression between 0.32 and 0.36.

With the extrapolated data, it was only possible to perform a meta-analysis on OS considering the RR, whose aggregate value was 0.36 95% CI: [0.25, 0.51], as expected, in favor of a much smaller number of events in the presence of high miR-195 expression.

These data are clearly statistically significant in favor of high expression; the risk of bias in the three included studies and between the studies was considered acceptable, and the TSA confirmed that there was an adequate number of patients with an adequate statistical power of the data. Grade assessment provided us with the quality of evidence deemed high.

The data obtained from the TGCA analysis, on the other hand, showed slightly worse values for HR (which is correlated time) in the course of the high expression of miR-195; these data do not necessarily deny the data obtained from the meta-analysis for the RR; in fact, even if the adverse events (death of the patient) are greater in the course of low expression, when these data are related as a function of time and therefore of the follow-up, the OS was superior to those with low expression.

The low number of studies conducted limits the results of this review that may have influenced the results of the meta-analysis through the publication bias; even the absence of HR values, but only RR may be a factor limiting the results of the review.

The studies included in this systematic review were type 2, in which the objective was to evaluate the specific association between specific factors and clinical outcomes, as reported in the prognosis research strategies (PROGRESS). The limitation of these type 2 studies is to divide the cohorts of “high” and “low” risk patients but do not consider the probability of individual patients to develop the outcome. In this context, a review with meta-analysis performed on type 2 studies can only precede studies on “prognostic model research”, which are type 3. At present, there are no studies on miR-195 and HNSCC in phase 3 yet [35]. 

## 5. Conclusions

In conclusion, even though this meta-analysis was limited, it can be stated that miR-195 is clearly downregulated in HNSCC carcinoma tissues, and furthermore, its low expression has excellent potential to be an independent prognostic survival biomarker for HNSCC. Consequently, exhaustive investigations of miRNA, for instance, investigations regarding the intercommunication among miRNAs and between miRNAs and other genes, the altered protein expression induced by miRNAs and site-specific miRNA expression profiling are prerequisites before future clinical trials of therapeutic applications are conducted.

## Figures and Tables

**Table 1:** The data extracted for the four articles included in the meta-analysis.

First Author, Data	Country	Study Design	Number of Patients (Male, Female); Staging (I–II, III–IV)	Smoking (Y, N,)	Follow-Up Max	Tumor Type/Tumor Site	Cut-off, Median.	miR	RR miR-195 Low and High Expression (OS, PFS, CSS, DFS, RFS)
[23] Shuang et al., 2017	China	Prospective	122 (80, 42); S (23, 99)	99, 23	60 months	LSCC	Median fold change (T/N = 0.436)	miR-195	OS: RR 0.358 (0.134–0.959) *p*= 0.041
[31] Ding and Qi, 2019	China	Prospective	182 (120, 62); S (130, 50)	/	60 months	LSCC	Average expression level 0.76 ± 0.38	miR-195	OS: RR 0.3616 * (0.2409–0.5428) *p* = 0.0001
[32] Jia et al., 2013	China	Prospective	81 (45, 36); S (48, 33)	/	48 months	TSCC	Median fold change (T/N = 0.652)	miR-195.	OS: RR 0.322 (0.120–0.865) *p* = 0.025

* The RR calculation was obtained by starting from the data expressed as a % of the events in the two groups (low and high expression of mir-195). Tongue squamous cell carcinoma (TSCC), S (Staging).

**Table 2:** Assessment of the risk of bias within the studies.

First Author, Data	Sample	Clinical Data	Marker Quantification	Prognostication	Statistics	Classical Prognostic Factors	Score
[23] Shuang et al., 2017	3	2	3	2	3	2	15
[31] Ding and Qi, 2019	3	2	3	2	3	2	15
[32] Jia et al.,2013	2	2	3	2	3	2	14

**Table 3:** Evaluation of GRADE pro GD.

Certainty Assessment							No. of Patients		Effect		Certainty
No. of Studies	Study Design	Risk of Bias	Inconsistency	Indirectness	Imprecision	Other Considerations	miR-195	Placebo	Relative (95% CI)	Absolute (95% CI)	
**mir-195 HNSCC**											
**3**	Randomized trials	Not serious	Not serious	Not serious	Not serious	Strong association	385	-/0	**RR 0.36** (0.25 to 0.51)	**0 fewer per 1.000** (from 1 fewer to 0 fewer)	⨁⨁⨁⨁High

**CI:** confidence interval; **RR:** risk ratio.

## Data Availability

Not applicable.

## Supplementary Materials

The following supporting information can be downloaded at: https://www.mdpi.com/article/10.3390/jpm13020275/s1, PFA the supplementary material file “cutt-off values in relation to the p-values”.
