# Supplementary material for: Is the Non-Coding RNA miR-195 a Biodynamic Marker in the Pathogenesis of Head and Neck Squamous Cell Carcinoma? A Prognostic Meta-Analysis
Source: J Pers Med. 2023 Jan 31;13(2):275. doi: 10.3390/jpm13020275 (PMC9963742; doi:10.3390/jpm13020275)
Supplement: Supplementary file 1 [file jpm-13-00275-s001.zip › jpm-2163429-supplementary materials.pdf]

"cutoff value" in relation to the "*p*-values":

13 0.462087469662889
13 0.425910482743201
13 0.504088415911796
13 0.596210102380106
13 0.664766293155924
13 0.62012607872158
13 0.709985741502504
13 0.653858326841281
13 0.7737775052278
13 0.896723044135075
13 0.814551772645132
13 0.721930581682705
13 0.675971732875581
13 0.632651085006734
13 0.746187241228819
13 0.851725290589604
13 0.824669097211427
14 0.778125904990256
14 0.732128123290961
14 0.719590729015675
14 0.838899621702853
14 0.918267121626508
14 0.89098742709557
14 0.818431244980301
14 0.909464501609736
14 0.951553331929768
14 0.857602276322061
14 0.981621572400742
14 0.927803284834511
14 0.992176572920455
14 0.912081327503292
14 0.954880069518734
15 0.894276012373776
15 0.96567293295303
15 0.969198709946474
15 0.891500903302076
15 0.959696615387007
15 0.928688534742065
15 0.865678329571579
15 0.921593813108529
15 0.968650112976812
15 0.983410312005387
15 0.960512633360418
15 0.91759972464082
16 0.949258124001711
16 0.953572032330212
16 0.973161694315339
16 0.910179456580133
16 0.80806753237727
16 0.829528282443271
16 0.90298786639434
16 0.994040949196432
16 0.915471846282425
16 0.978371292464373
16 0.914689390650638
16 0.797414907634396
16 0.7461939478993
16 0.805486419155215
16 0.849656835263971
16 0.895499894222458
16 0.849875091205061

16 0.938820158311509
17 0.952083730288526
17 0.956993318339619
17 0.98821653898596
17 0.976817487901753
17 0.946185394278755
17 0.896891433331776
17 0.997025756658378
17 0.923419865751729
17 0.972884613902202
17 0.984025882750667
17 0.916790902446418
17 0.860141622809037
17 0.783951016976704
17 0.832789511996894
17 0.800110830514341
17 0.753280984200387
17 0.813439562394325
17 0.878435399534828
17 0.947625109066677
17 0.997779098193164
17 0.892074927157621
17 0.979162968684596
17 0.873678102527018
17 0.90347072330321
17 0.814885013811697
17 0.736943356835522
17 0.761902745269021
17 0.831570069090017
18 0.762866000773705
18 0.769272708561366
18 0.69571603664708
18 0.715378753685047
18 0.716421556458804
18 0.633354628883582
18 0.540248263108171
18 0.615091656432987
18 0.543402945473439
18 0.460554499153545
18 0.472921909000327
18 0.49028968635386
18 0.432927472829214
18 0.474174552829142
18 0.49292352006808
18 0.553533755264755
18 0.591542061212404
18 0.502721907615822
19 0.471192718949201
19 0.499090012110265
19 0.537404061628902
19 0.583109192374418
19 0.6481026513395
19 0.727508484749359
19 0.809674721937089
19 0.872721988995735
19 0.934860135147672
19 0.989563173882055
19 0.885264176106509
19 0.970171203450557
19 0.864353249823169
19 0.788102570148522
19 0.804651278348997
19 0.850116376002514

19 0.897521999198708
19 0.982329226930959
19 0.972607578407841
19 0.965487938174857
19 0.998722911857846
19 0.960016549570145
19 0.884835267340147
20 0.994208509462984
20 0.92458153382108
20 0.815701270093897
20 0.885720225892667
20 0.830892092184974
20 0.873798028750686
20 0.958090580410115
20 0.977356116811371
20 0.94253093629019
20 0.878417817550652
20 0.831964732753656
20 0.750388306661412
20 0.79356500170537
20 0.713187343720994
20 0.646861004660805
20 0.627229250284711
20 0.582721306200946
20 0.512290534132944
20 0.503575694326855
20 0.438480589779543
21 0.488264734478728
21 0.424260390009123
21 0.373575844133433
21 0.332179241394987
21 0.294762672653427
21 0.354068018989371
21 0.4318438837083
21 0.372231370264408
21 0.330285611736803
21 0.302913238369919
21 0.276308690902306
22 0.253995127667951
22 0.312785120952093
22 0.28779768814318
22 0.255295335935838
22 0.223155581753597
22 0.20161515423226
22 0.172521578264899
22 0.169364444082335
22 0.147236574379316
22 0.170548359499805
22 0.185989127755908
22 0.203681609329426
22 0.17463264268014
23 0.153983420892361
23 0.17790425003208
23 0.159682135773768
23 0.199569279437766
23 0.170818901084265
23 0.217332889175328
23 0.270557147814715
23 0.239669424839207
23 0.198994273396997
23 0.247715511473687
23 0.219869085551346
23 0.235902674559757

23 0.20706993546064
23 0.185826737927498
24 0.170318525419844
24 0.164557565805784
24 0.143837319274664
24 0.129381971495174
24 0.157702420098343
24 0.127715224051276
24 0.107568243608956
24 0.0853687892610103
24 0.0773078800768783
24 0.102761205539523
24 0.128701574863768
25 0.157444647281186
25 0.194789174176338
25 0.164930550122968
25 0.212118686704643
25 0.255693544752785
25 0.309406939640458
25 0.330932360874808
25 0.388433597039747
26 0.361389268768912
26 0.319886095226998
26 0.279455525263102
26 0.340672755562005
26 0.403162826800084
26 0.475210612333572
26 0.447421687519076
26 0.534852968544752
26 0.463901435514211
26 0.398229041537374
26 0.466777044626207
26 0.453653835612638
26 0.444195486884586
26 0.484496468583544
26 0.568220798330985
26 0.629523151808852
26 0.692337456769235
26 0.661992954797019
26 0.739370671814742
27 0.674427419802945
27 0.781823701759715
27 0.696565285747537
27 0.62001678079817
27 0.570325376179685
27 0.564135929245461
27 0.489301279794835
27 0.549902027765911
27 0.475683777117463
27 0.558716589947049
27 0.516079574444971
27 0.505641123138214
27 0.502550280249021
27 0.571977387638614
28 0.667597518413323
28 0.618209527664
28 0.705632482127391
28 0.633373755646696
28 0.561202649683282
28 0.633581556178017
28 0.724051534347813
28 0.721331799163669
28 0.689703854330811

28 0.77482218613734
29 0.722016329388859
29 0.79022660487407
29 0.866894484444422
29 0.928067971147883
29 0.874490265091104
29 0.81164545952196
29 0.905259722540206
29 0.989417373308441
29 0.922928052797476
29 0.875896128511295
29 0.999680278699658
29 0.895894716503247
